# Host MicroRNA-217 Promotes White Spot Syndrome Virus Infection by Targeting *Tube* in the Chinese Mitten Crab (*Eriocheir sinensis*)

**DOI:** 10.3389/fcimb.2017.00164

**Published:** 2017-05-04

**Authors:** Ying Huang, Keke Han, Wen Wang, Qian Ren

**Affiliations:** ^1^Jiangsu Key Laboratory for Biodiversity and Biotechnology and Jiangsu Key Laboratory for Aquatic Crustacean Diseases, College of Life Sciences, Nanjing Normal UniversityNanjing, China; ^2^Co-Innovation Center for Marine Bio-Industry Technology of Jiangsu ProvinceLianyungang, China

**Keywords:** *Eriocheir sinensis*, WSSV, miR-217, toll signaling pathway, *Tube*, antimicrobial peptides

## Abstract

MicroRNAs (miRNAs), a group of small molecule non-encoding RNAs, are key post-transcriptional regulators of gene expression that are implicated in many biological processes. In the current study, miR-217 from *Eriocheir sinensis* was selected for studying its roles during host–virus interaction. Overexpression or silencing of miR-217 led to considerable effects on white spot syndrome virus (WSSV) replication, implying that miR-217 played a positive role in WSSV infection. In insect High Five cells, miR-217 significantly inhibited *Tube* gene expression by binding to the 3′-untranslated region of the *Tube*. Overexpression of miR-217 in crab led to downregulation of *tube* expression. Knockdown of *Tube in vivo* led to significant enhancement of WSSV infection and inhibited the expression of five *antimicrobial peptide* (*AMP*) genes (*Anti-lipopolysaccharide factor ALF1, ALF2, ALF3*; *Crustin Crus1, Crus2*) in WSSV-challenged crabs. Overexpression of miR-217 also led to downregulation of these *AMP* genes in WSSV-challenged crabs. Our results showed that host miRNA played positive roles in virus infection by regulation of host *tube* gene, which is the key component of Toll signaling pathway.

## Introduction

MicroRNAs (miRNAs) are a class of small non-coding RNAs that are found in diverse eukaryotic organisms (Carrington and Ambros, 2003). MicroRNAs are endogenous, 18 to 26 nucleotides (nt) long, and are processed from the hairpin transcripts cleaved by two RNase III enzymes, namely, Drosha and Dicer (Bernstein et al., 2001; Bartel, 2004). The mature miRNA strand is incorporated into the RNA-induced silencing complex, wherein mRNA degradation or protein translation repression is controlled mainly through acting on the 3′-untranslated region (UTR) of target mRNAs (Schwarz et al., 2003; Meister and Tuschl, 2004; Bagga et al., 2005). Since *lin-4* and *let-7*, the first miRNAs, were discovered in *Caenorhabditis elegans* (Lee et al., 1993; Reinhart et al., 2000), numerous miRNAs have been found to be involved in the regulation of diverse biological processes, including development, differentiation, apoptosis, oncogenesis, and immune activation (Ambros, 2004; Lecellier et al., 2005; Pedersen et al., 2007). Host miRNAs are believed to be key regulators of virus–host interactions (Triboulet et al., 2007; Umbach and Cullen, 2009). For example, human miR-125a has been shown to suppress the replication of hepatitis B virus by binding the viral transcript encoding the surface antigen and interfering with its expression (Mosca et al., 2014). MiR-155 inhibits Japanese encephalitis virus replication and negatively modulates innate immune responses in JEV-infected human microglial cells (Pareek et al., 2014). Two host cellular miRNAs, namely, miR-320a and miR-140, were found to inhibit mink enteritis virus infection into feline kidney cells by repression of its receptor, feline transferrin receptor (Sun et al., 2014). Host miR-26a suppresses the replication of porcine reproductive and respiratory syndrome virus by upregulating the expression of type I interferon, as well as the IFN-stimulated genes MX1 and ISG15 (Li L. et al., 2015). However, a small amount of information is available regarding the pathways mediated by host miRNAs in invertebrates.

Invertebrates lack antibodies and complements; however, they can initiate a rapid and effective innate immune response against pathogenic organisms (Uematsu and Akira, 2008). Recognizing invading microbial pathogen, which is conducted by a set of so-called pattern recognition receptors (PRRs), is an essential step in innate immune responses (Bischoff et al., 2004). Toll-like receptors or Tolls, which initiate innate immune responses by sensing pathogen-associated molecular patterns (Kawai and Akira, 2010) and then activating evolutionarily conserved Toll signal transduction (Imler and Hoffmann, 2001), are the most extensively studied PRRs. Toll signaling occurs through several adaptor proteins, such as myeloid differentiation factor 88 (MyD88), Tube, and Pelle (Valanne et al., 2011). Upon ligand binding, MyD88 recruits the activated Toll receptor and the cytosolic adaptor Tube to transfer Toll signaling. Then, Pelle is recruited to the vicinity of Tube, forming a trimeric complex (MyD88-Tube-Pelle) mediated by the death domain of Tube. Finally, the signal is transmitted to the Dorsal/Cactus complex that regulates the Toll-dependent gene expression, such as *antimicrobial peptides* (*AMPs*), as well as a substantial number of other innate immune responsive genes (Belvin and Anderson, 1996; Lemaitre and Hoffmann, 2007; Valanne et al., 2011). The adaptor Tube is an essential component of the Toll signaling pathway. The *Drosophila melanogaster* Tube not only participates in dorsoventral axis formation during development but is also involved in regulating diverse downstream signaling as well as the response against gram-positive bacteria and fungi (Lemaitre et al., 1996; Wang et al., 2006). The mud crab *Scylla paramamosain*, SpTube, together with SpPelle and SpMyD88, may form a trimeric complex in the Toll signaling cascade and are shown to be involved in the immunity against gram-positive and gram-negative bacteria (Li et al., 2013). *EsTube* might play important roles in the innate immune response to exogenous pathogenic stimulation and the development of the gonads in the Chinese mitten crab, *Eriocheir sinensis* (Yu et al., 2014). Therefore, Tube may have a vital role in initiating and activating the immune system. However, the host–virus interaction between *Tube* and miRNAs is not addressed at present.

White spot syndrome virus (WSSV) is one of the most virulent agents threatening the shrimp culture industry worldwide (Escobedo-Bonilla et al., 2008). Recently, one report showed that WSSV also caused serious disease, thereby threatening the Chinese mitten crab aquaculture industry (Ding et al., 2015). Given the breadth of miRNA-mediated regulation of immunity, the role of host miRNAs in WSSV infection is of significant interest. Herein, the crab miR-217 targeting *Tube* was characterized in this study. Our findings indicated that miR-217 first directly targeted *Tube*. Then, it regulated the expression of *AMP* genes. Finally, miR-217 promoted the WSSV infection in *E. sinensis*.

## Materials and methods

### Crab culture and WSSV challenge

Approximately 40 g of *E. sinensis* was raised in a group of 30 individuals at room temperature. Three crabs from each group were randomly selected for the PCR detection of WSSV with WSSV-specific primers (5′-TATTGTCTCTCCTGACGTAC-3′ and 5′-CACATTCTTCACGAGTCTAC-3′) to ensure that the crabs were virus-free before experiments. Then, the virus-free crab was infected with 100 μl WSSV solution for each crab at 10^5^ copies/ml by intramuscular injection. At different times post-infection (0, 2, 4, 6, 12, 24, 36, 48, 60, and 72 h), the hemolymph was collected from treated crabs and subsequently centrifuged at 2000 rpm at 4°C for 10 min to harvest the hemocytes. Other tissues (heart, hepatopancreas, gills, muscles, intestines, nerves, and eyestalk) from the five untreated crabs were also collected for later use.

### Detection of crab miR-217 using real-time PCR

Total RNAs were extracted from the preceding issues mentioned using the mirVana miRNA isolation kit following the instructions of the manufacturer (Ambion, USA). cDNA for miRNA qRT-PCR was then synthesized with All-in-one™ miRNA First-Strand cDNA Synthesis Kit (GeneCopoeia™, USA) according to the protocol of the manufacturer. Quantitative real-time PCR was carried out according to the protocol of All-in-one™ miRNA qRT-PCR Detection Kit (GeneCopoeia™, USA) using the miR-217 special primer (5′-CGCTACTGCATCAGGAACTGA-3′). Glyceraldehyde-3-phosphate dehydrogenase (*GAPDH*) was used as a control. The first-strand cDNA synthesis was obtained by using the PrimeScript™ RT reagent Kit (Perfect Real Time) (Takara, Dalian, China), and qRT-PCR was carried out using 2 × SYBR Premix Ex Taq kit (Takara, Japan) with *GAPDH*-specific primers (5′-CTGCCCAAAACATCATCCCATC-3′ and 5′-CTCTCATCCCCAGTGAAATCGC-3′).

### Silencing or overexpression of miR-217 in crab

The anti-miRNA-217 oligonucleotide (AMO-miR-217: 5′-ATCCAATCAGTTCCTGATGCAGTA-3′, 10 nM) and WSSV (10^5^ copies/ml) were coinjected into crab to silence the expression of miR-217. As a control, the sequence of AMO-miR-217 was scrambled, yielding AMO-miR-217-scrambled (5′-CACTAAGTGGATGCTAAACTTCTC-3′). The AMO-miR-217-scrambled (10 nM) and WSSV (10^5^ copies/ml) were included in the injections. The oligonucleotides were sequentially injected into the same crab twice at a 16 h interval to avoid the degradation of AMO-miR-217 or AMO-miR-217-scrambled in crab. WSSV alone (10^5^ copies/ml) and phosphate buffer saline (PBS) were also injected into crab. At 24, 36, and 48 h after the last injection, the hemolymph specimens selected at random from three crabs were mixed and subjected to subsequent analysis.

The mimic of miR-217 (5′-UACUGCAUCAGGAACUGAUUGGAU-3′) was synthesized with an *in vitro* transcription T7 kit for siRNA synthesis (Takara, Japan). The sequence of miR-217 was scrambled to generate the control miR-217-scrambled (5′-CUCAAGGAUGCGUUAAGGUUACUA-3′). miRNA (20 μg) and WSSV (10^5^ copies/ml) were coinjected into healthy crabs to overexpress miR-217. At 16 h after the coinjection, the miRNA (20 μg) (100 μl /crab) was injected into the same crab. As controls, miR-217-scrambled, WSSV (10^5^ copies/ml), and PBS were included in the injections. At different time points after the last injection, the hemolymph specimens from three crabs were selected. All the preceding assays described were biologically repeated for three times.

### Quantitative analysis of WSSV copies by real-time PCR

The genomic DNA was extracted to quantify the WSSV copies in crab. qRT-PCR was performed with WSSV-specific primers (5′-TTGGTTTCATGCCCGAGATT-3′ and 5′-CCTTGGTCAGCCCCTTGA-3′) and a TaqMan probe (5′-FAM-TGCTGCCGTCTCCAA-TAMRA-3′). The plasmid containing a DNA fragment of 1,400 bp from the WSSV genome was used as the internal standard of real-time PCR (Liu et al., 2009). The 25 μl PCR reaction solutions contained 12.5 μl of the Premix ExTaq (Takara, Japan), 0.5 μl of 10 μM primers each, 1 μl of 10 μM TaqMan fluorogenic probe, 1 μl of DNA template, and 9.5 μl of distilled water. The pre-denaturalization stage of the PCR program was 95°C for 1 min, followed by the amplification stage comprising 40 cycles of 95°C for 30 s, 52°C of 30 s, and 72°C for 30 s.

### Prediction and identification of target genes of miR-217 in crab

Owing to the insufficient crab genome sequence, the assembled crab EST sequences (unpublished) were used to predict the target genes of miR-217 in crab. Three computational algorithms, including TargetScan 5.1 (http://www.targetscan.org), miRanda (http://www.microrna.org/), and Pictar (http://pictar.mdc-berlin.de/), were conducted. The data predicted by the three algorithms were overlapped and calculated.

### Plasmid constructions

To explore the interaction between miR-217 and its target gene *Tube*, the enhanced green fluorescent protein (*EGFP*) gene was amplified from the pEGFP vector (BD Biosciences, USA) using *EGFP* forward and reverse primers (5′-AAGAGCTCGGATCCCCGGGTAC-3′ and 5′-AATCTAGAGTCGCGGCCGCTTTA-3′) and cloned into the pIZ/V5-His vector (Invitrogen, USA) to produce a control EGFP construct. Subsequently, the *Tube* 3′UTR was cloned downstream of the *EGFP* gene using the XbaI and SacII restriction sites. The primers used for the amplification of *Tube* 3′UTR were 5′-GCTCTAGATGATGGCTGCATTCACTTGG-3′ and 5′-TCCCCGCGGTAATTACATTGATGATATCA-3′. The sequence of *Tube* 3′UTR complementary to the miR-217 seed sequence (ATGCAGT) was randomly mutated to CGTACTG, generating the EGFP-ΔTube construct. The amplification of ΔTube 3′UTR was conducted by PCR with sequence-specific primers 5′-GGATAGAAAATGTGTCCACGTACTGACCTCATGG-3′ and 5′-ATCTTCTTACCATGAGGTCAGTACGTGGACACATT-3′. Recombinant plasmids were confirmed by sequencing.

### Cell culture, transfection, and fluorescence assays

Insect High Five cells (Invitrogen) were cultured at 28°C in Express Five serum-free medium (SFM, Invitrogen) containing L-glutamine (Invitrogen). The cells were cotransfected with the EGFP, EGFP-Tube, or EGFP-ΔTube plasmid and the synthesized miR-217 (miR-217-mimic) using the Cellfectin transfection reagent (Invitrogen) according to the protocol of the manufacturer. The concentrations of miR-217-mimic and the plasmid were 200 nM/well and 400 ng/well, respectively. As a control, the control miRNA was included in the cotransfection. All the miRNAs were synthesized by Shanghai GenePharma Co., Ltd. (Shanghai, China). At 48 h after cotransfection, the EGFP fluorescence of the cells was measured by a Flex Station II microplate reader (Molecular Devices, USA) at 490/510 nm of excitation/emission (Ex/Em).

### RNAi assays in crab *in vivo*

Based on the sequences of crab *Tube* gene, siRNA (Tube-siRNA: 5′-CCCAAACGGCAACCCUCAU-3′) was synthesized according to the design rule for siRNA. The sequence of siRNA was randomly scrambled to generate the control siRNA, Tube-siRNA-scrambled (5′-UCACCUACGCACCACACAG-3′). To silence *Tube* expression, Tube-siRNA (20 μg) and WSSV (10^5^ copies/ml) were coinjected into each crab. Sixteen hours later, the siRNA was injected into the same crab at 20 μg/crab. WSSV (10^5^ virus copies/ml) alone was used as a positive control. At different times (24, 36, and 48 h) after the injection of siRNA, the crab hemocytes was collected and stored at −80°C for later use.

### Quantification of mRNA with real-time PCR

The mRNA levels of *Tube* and *AMP* genes in crab were determined using qRT-PCR. The *Tube* primers were 5′-ATTGTGCTGCTGGAGTTGCTGAC-3′ and 5′-CATCGTCGGTCGCTTCTTCTTGG-3′. *Anti-lipopolysaccharide factor 1* (*ALF1*), *ALF2, ALF3, Crustin 1* (*Cru1*), and *Crustin 2* (*Cru2*) were detected with sequence-specific primers (ALF1-RT-F: 5′-GACGCAGGAGGATGCTAAC-3′, and ALF1-RT-R: 5′-TGATGGCAGATGAAGGACAC; ALF2-RT-F: 5′-GACCCTTTGCTGAATGCTTGA-3′, and ALF2-RT-R: 5′-CTGCTCTACAATGTCGCCTGA-3′; ALF3-RT-F: 5′-ACGAGGAGCAAGGAAAGAAAG-3′, and ALF3-RT-R: 5′-TTGTGCCATAGACCAGAGACTT-3′; Crus1-RT-F: 5′-GCTCTATGGCGGAGGATGTCA-3′, and Crus1-RT-R: 5′-CGGGCTTCAGACCCACTTTAC-3′; Crus2-RT-F: 5′-GCCCACCTCCCAAACCTAT-3′, and Crus2-RT-R: 5′-GCAAGCGTCACAGCAGCACT-3′). Crab *GAPDH* was used as the internal reference. The 2^−ΔΔCT^ method was used to calculate the relative fold-change of the mRNA expression level (Livak and Schmittgen, 2001).

### Statistical analysis

Numerical data were analyzed using one-way analysis of variance to calculate the mean and standard deviation of triplicate assays. Unpaired sample *t*-test was conducted, and the differences were considered significant if *P* < 0.05 or *P* < 0.01.

## Results

### Effects of miR-217 on virus infection in crab

**Figure 1:**
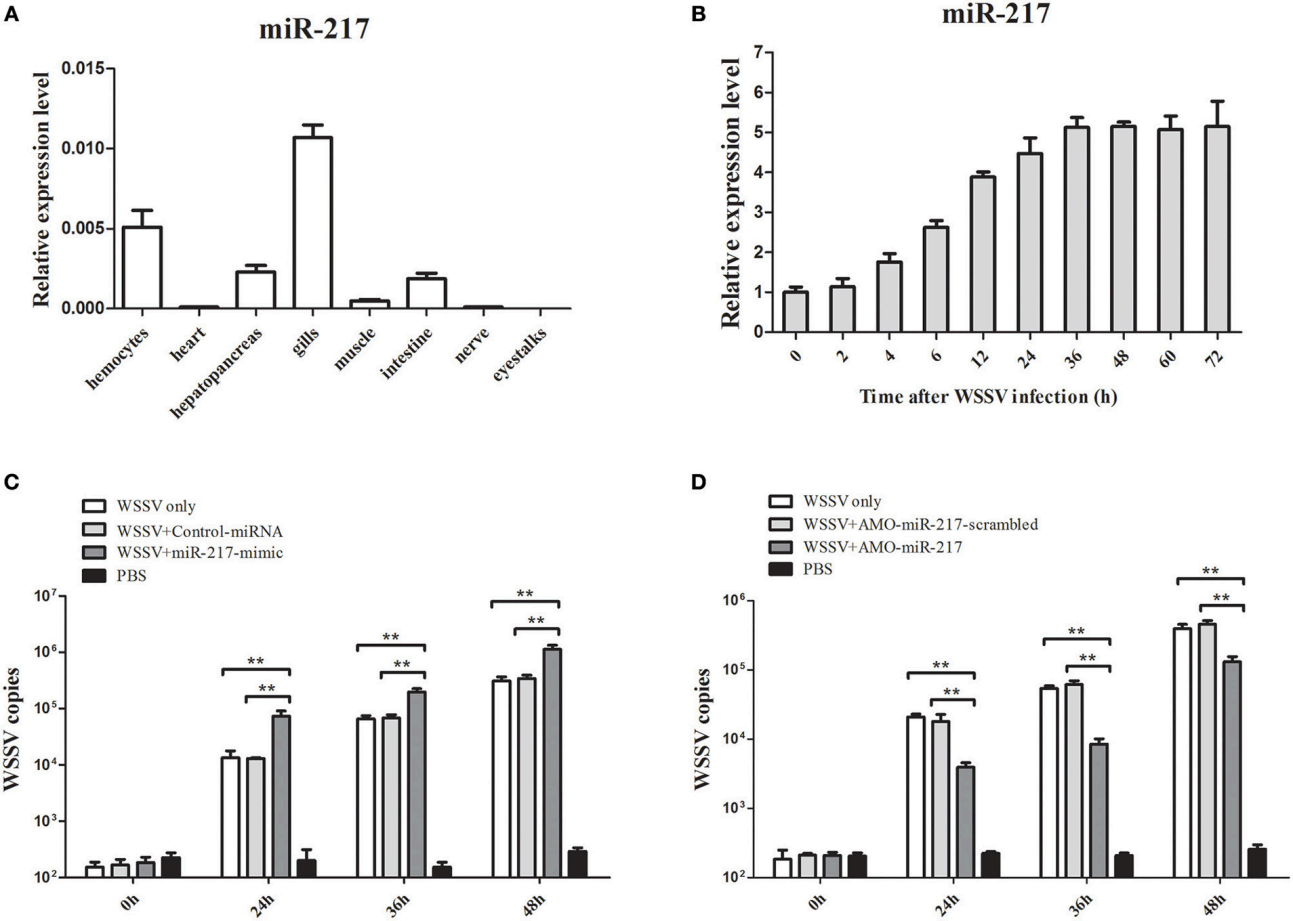
**Effects of miR-217 on virus infection in crab. (A)** Tissue distribution of miR-217 transcripts measured by quantitative real-time PCR. **(B)** The expression profiles of miR-217 by qRT-PCR after WSSV challenge. Total miRNA was extracted from crab hemocytes. Crab *GAPDH* was used as the reference gene for internal controls. **(C)** Effect of miR-217 overexpression on the virus infection. MiR-217-mimic or control miRNA was injected into WSSV-infected crab. At different times after treatments, the crab hemocytes were subjected to qRT-PCR to determine the WSSV copies. **(D)** Influence of miR-217 silencing on the virus infection. The crabs were injected with AMO-miR-217 to knock down the expression of miR-217 and challenged with WSSV. The AMO-miR-217-scrambled, WSSV only and PBS only were used as controls. At various time post-infection, the WSSV copies in crab were evaluated. Data presented were the mean ± standard deviation of three independent experiments. Asterisks indicate significant differences (^**^p < 0.01) between treatments.

MiR-217 expression in several tissues was performed by qRT-PCR (Figure 1A). *GAPDH* was used as a reference. MiR-217 was mainly expressed in gills and hemocytes, with a relatively low expression level in the hepatopancreas, intestine, and muscle. The expression changes of miR-217 in WSSV-challenged crab were detected to explore the relationship between miR-217 and WSSV infection. From the result of qRT-PCR, the WSSV infection led to a significant increase of miR-217 expression level in the hemocytes of crab (Figure 1B). MiR-217-silenced or overexpressed crabs were analyzed with the assays of the WSSV copies to further evaluate the role of miR-217 in the virus infection. The data presented that the WSSV copies were significantly increased by the miR-217 overexpression and significantly decreased by the miR-217 silencing compared with the control (Figures 1C,D), indicating that miR-217 played a positive role in regulation of the WSSV infection.

### Interaction between host miR-217 and host *Tube* gene *in vitro*

**Figure 2:**
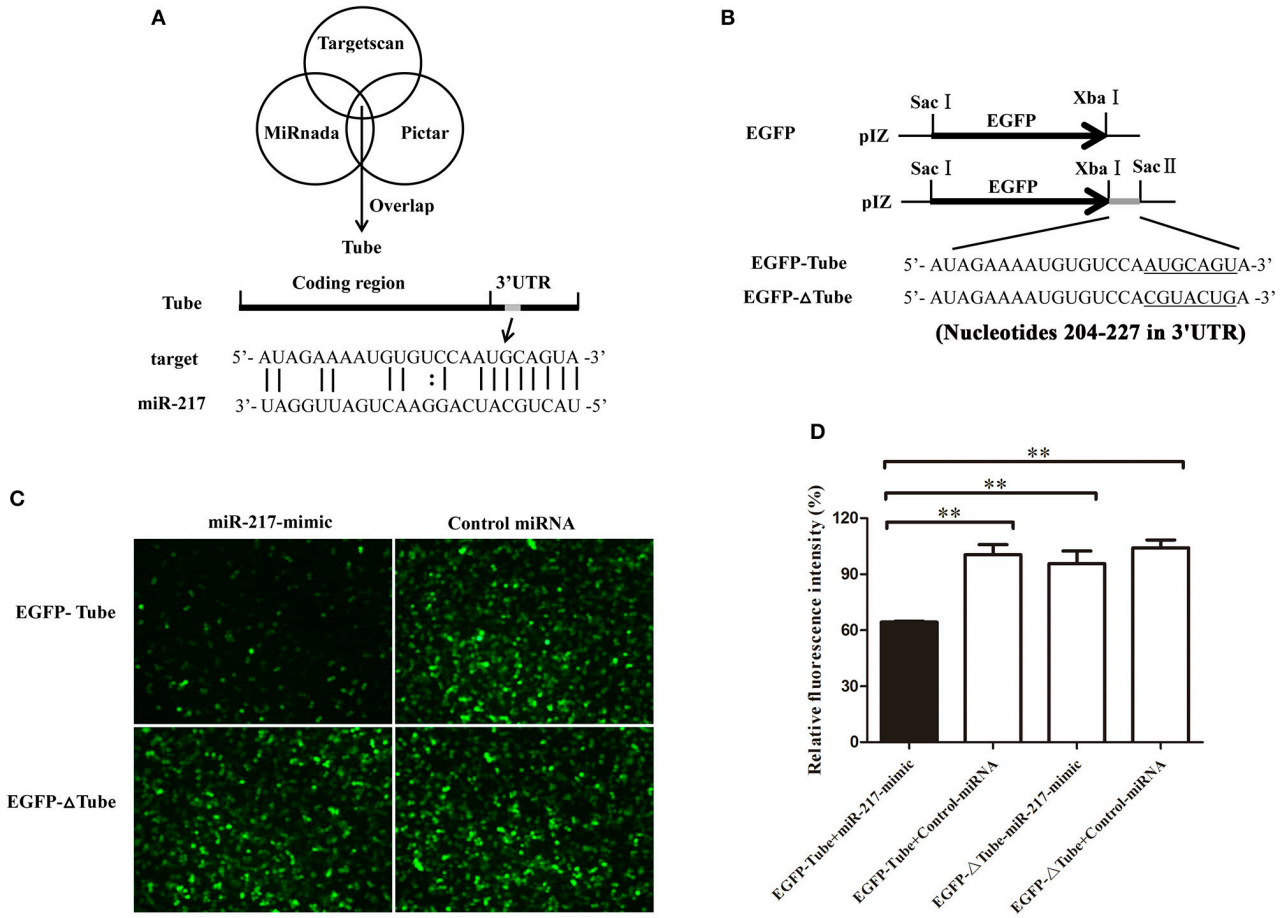
**Interaction between host miR-217 and host *Tube* gene. (A)** The region of *Tube* 3′UTR sequence targeted by miR-217 predicted with three algorithms. **(B)** The construction of the wild-type and mutated 3′UTRs of *Tube* gene. The *Tube* sequence targeted by the miR-217 seed sequence and the mutated sequence were underlined. **(C)** Interaction between host miR-217 and *Tube* gene in insect cells. The insect High Five cells were cotransfected with miR-217-mimic and different plasmids (EGFP-Tube, or EGFP-ΔTube). To act as a control, the control miRNA was included in the transfections. The constructs EGFP-Tube, or EGFP-ΔTube contained the wild-type and mutant *Tube*, respectively. At 48 h after transfections, fluorescent images were obtained. **(D)** The relative fluorescence intensity of cells. The values refer to the means ± standard deviation of triplicate assays. ^**^Statistically significant differences (*p* < 0.01).

Growing evidence has indicated that host miRNAs play important roles in host–virus interactions in invertebrates. In this study, the prediction results indicated that the crab miR-217 could target the host *Tube* (GenBank Accession No. KC011815), an important adaptor protein of the Toll pathway (Figure 2A). MiR-217-mimic and the plasmid EGFP-Tube were cotransfected into High Five cells because a crab cell line was unavailable (Figure 2B) to evaluate the direct interaction between miR-217 and *Tube*. The results revealed that the fluorescence intensity in the cotransfected cells was significantly weaker than that in the controls, showing that miR-217 targeted the *Tube* gene (Figures 2C,D). EGFP-ΔTube was constructed to investigate the specificity of the interaction between miR-217 and *Tube* 3′UTR. The result showed that miR-217-mimic had no effect on the expression of EGFP in miR-217-mimic and EGFP-ΔTube cotransfected cells, showing that miR-217 seed sequence was required to target the *Tube* 3′UTR.

### Interaction between miR-217 and *Tube* gene *in vivo*

**Figure 3:**
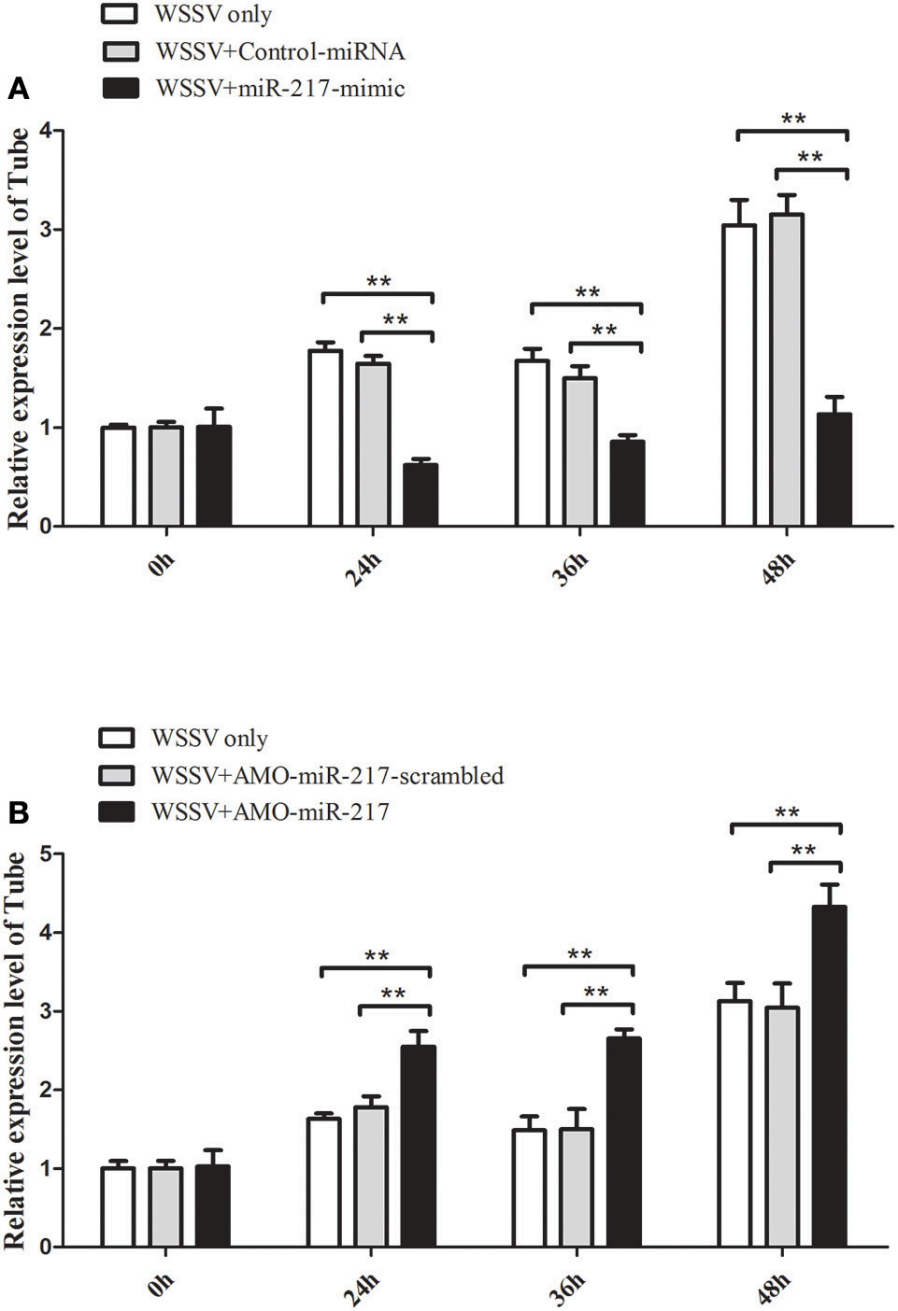
**Direct interaction between miR-217 and *Tube* gene *in vivo*. (A)** Influence of miR-217 overexpression on the expression of *Tube* gene. Either miR-217-mimic or control RNA was injected into WSSV-infected crab. At 24, 36, and 48 h after infection, the crab hemocytes were collected and subjected to examine the expression of *Tube* with qRT-PCR. *GAPDH* was used as a control. **(B)** Effect of miR-217 silencing on the expression of *Tube*. AMO-miR-217 or AMO-miR-217-scrambled was injected into WSSV-infected crab to knock down the miR-217 expression. At different times post-infection, the expression of *Tube* was detected with qRT-PCR. The values referred to the means ± standard deviation of triplicate assays. Statistically significant differences between treatments are indicated by asterisks (^**^p < 0.01).

MiR-217-mimic was injected into WSSV-infected crab to reveal the *in vivo* interaction between miR-217 and *Tube* gene in crab. The results indicated that overexpression of miR-217 led to a significant decrease in the transcript levels of the *Tube* gene at 24, 36, and 48 h post-infection (Figure 3A). AMO-miR-217 was injected into WSSV-infected crab to inhibit the expression of endogenous miR-217. The results showed that crab *Tube* expression was enhanced by AMO-miR-217 (Figure 3B). These results showed that changes in miR-217 levels could alter the expression of the host gene *Tube* in crab *in vivo*.

### Roles of *Tube* in crab–virus interaction

**Figure 4:**
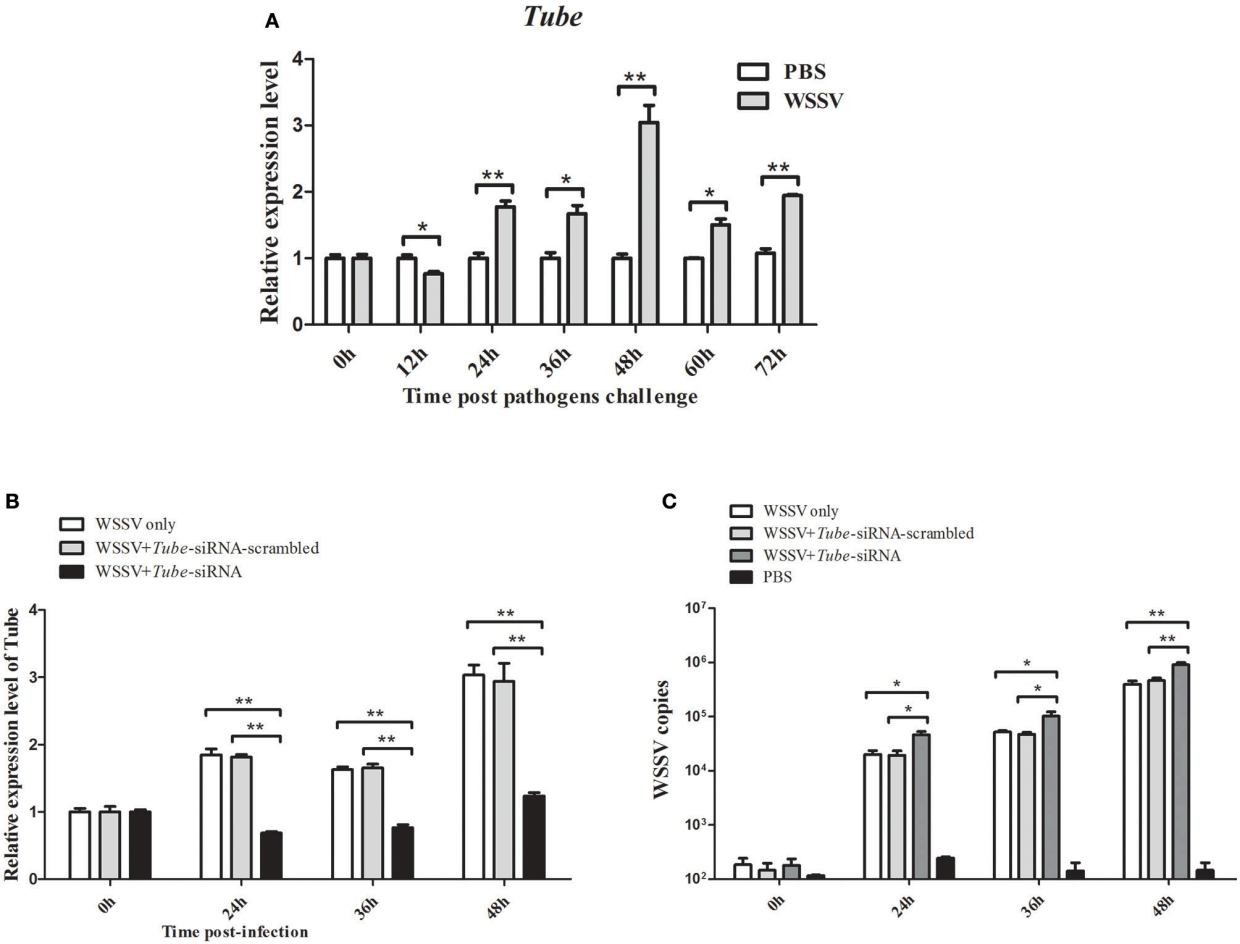
**Effects of *Tube* on virus infection in crab. (A)** Analysis of *Tube* expression in hemocytes of WSSV injected crabs by qRT-PCR at 0, 12, 24, 36, 48, 60, and 72 h post-injection. PBS was as control. **(B)** Silencing of the *Tube* expression in crab. WSSV and/or Tube-siRNA were injected into crab. At various time (24, 36, and 48 h) post-infection, the crab hemocytes tissues were collected and subjected to detect the expression of *Tube* with qRT-PCR. **(C)** The detection of WSSV copies. Upon the condition where *Tube* was silenced, the crab were collected and subjected to qRT-PCR to monitor the WSSV replication. The data were representatives of three independent experiments. The significant differences between treatments were indicated (^*^p < 0.05; ^**^p < 0.01).

QRT-PCR was used to analyze the *Tube* mRNA level after WSSV infection. At 24, 36, 48, 60, and 72 h in response to WSSV infection, the crab *Tube* was significantly upregulated (Figure 4A). The expression of the crab *Tube* gene was silenced by sequence-specific siRNA, followed by the examination of WSSV copies to further examine the roles of *Tube* in virus infection. The qRT-PCR result showed that the expression of *Tube* was repressed by the gene-specific siRNA from 24 h to 48 h after siRNA injection (Figure 4B). The mutation-siRNA had no effect on the expression of *Tube*. The data indicated that the *Tube*-siRNA was highly sequence specific, which could silence the *Tube* gene in crab *in vivo*. When the *Tube* gene had been silenced by *Tube*-siRNA, the numbers of WSSV genome copies/mg in crab hemocytes significantly increased compared with the control (WSSV only and *Tube*-siRNA-scrambled) (Figure 4C). The data indicated that *Tube* gene played important roles in WSSV replication.

### *AMP* genes expression are regulated by *Tube*

**Figure 5:**
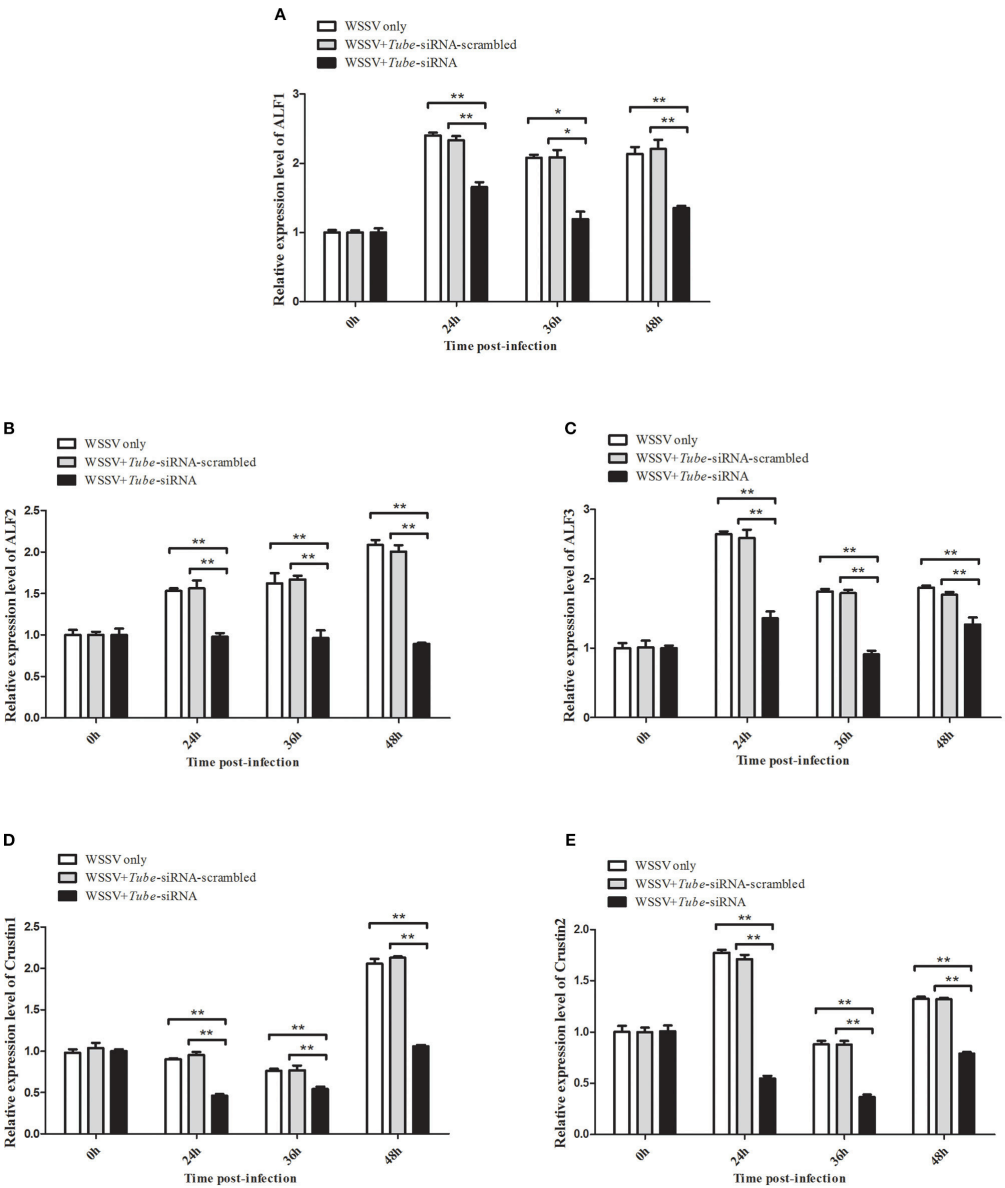
**Interaction between *Tube* gene and its downstream *AMP* genes**. The WSSV-infected crabs were injected with *Tube*-siRNA to silence the expression of *Tube*, followed by the quantification of *ALF1* **(A)**, *ALF2* **(B)**, *ALF3* **(C)**, *Crus1* **(D)**, or *Crus2* **(E)** mRNAs using qRT-PCR. The results are shown as mean and standard deviation from three independent experiments (^*^p < 0.05; ^**^p < 0.01).

*AMP* genes are important effectors of the Toll pathway in invertebrates. The expression of *Tube* was knocked down in crab to reveal the relationship between *Tube* and *AMP* genes *ALF1* (Accession No. DQ793214), *ALF2* (GU014699), *ALF3* (HQ850572.1), *Crus1* (GQ200832), and *Crus2* (GQ200833). Under the conditions wherein the *Tube* gene expression was silenced, five *AMP* gene expression levels were detected by qRT-PCR. The results are shown in Figure 5, wherein the mRNA transcriptions of *AMPs* were increased by different degrees of magnitude after the WSSV challenge. However, the expression levels of these *AMPs* were significantly decreased in the *Tube*-siRNA experimental group compared with the controls (*Tube*-siRNA-scrambled, WSSV only). Therefore, *ALF1, ALF2, ALF3, Crus1*, and *Crus2* are possibly the downstream genes of *Tube*.

### *AMP* genes expression are regulated by miR-217

**Figure 6:**
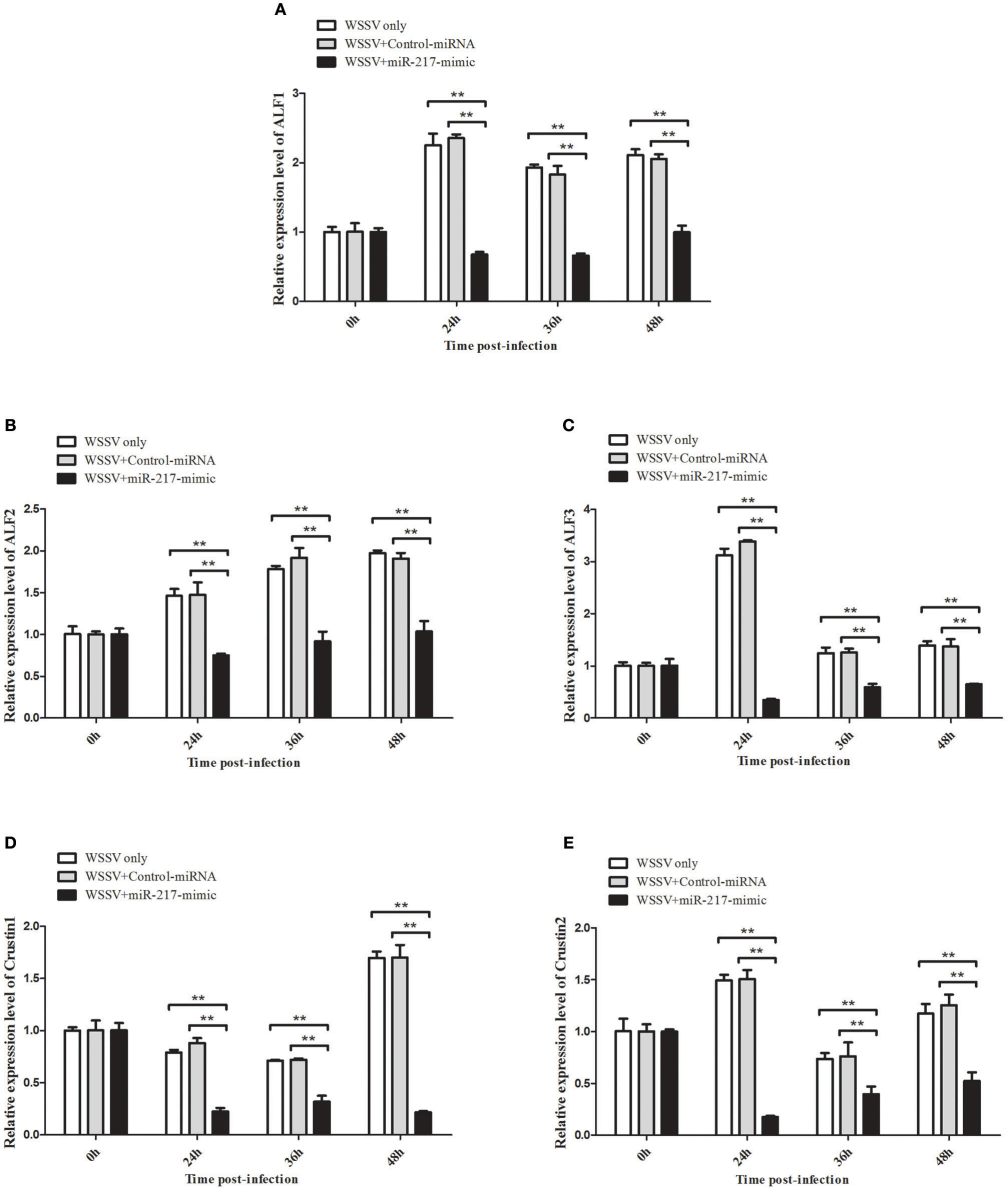
**Effects of miR-217 overexpression on the transcriptions of five *AMPs in vivo***. MiR-217-mimic or control miRNA was injected into WSSV-infected crab. As a control, WSSV only was included in the injections. At 24, 36, and 48 h after injection, the mRNA levels of *ALF1* **(A)**, *ALF2* **(B)**, *ALF3* **(C)**, *Crus1* **(D)**, or *Crus2* **(E)** were quantified by qRT-PCR. Statistically significant differences between treatments are represented with asterisks (^**^p < 0.01).

**Figure 7:**
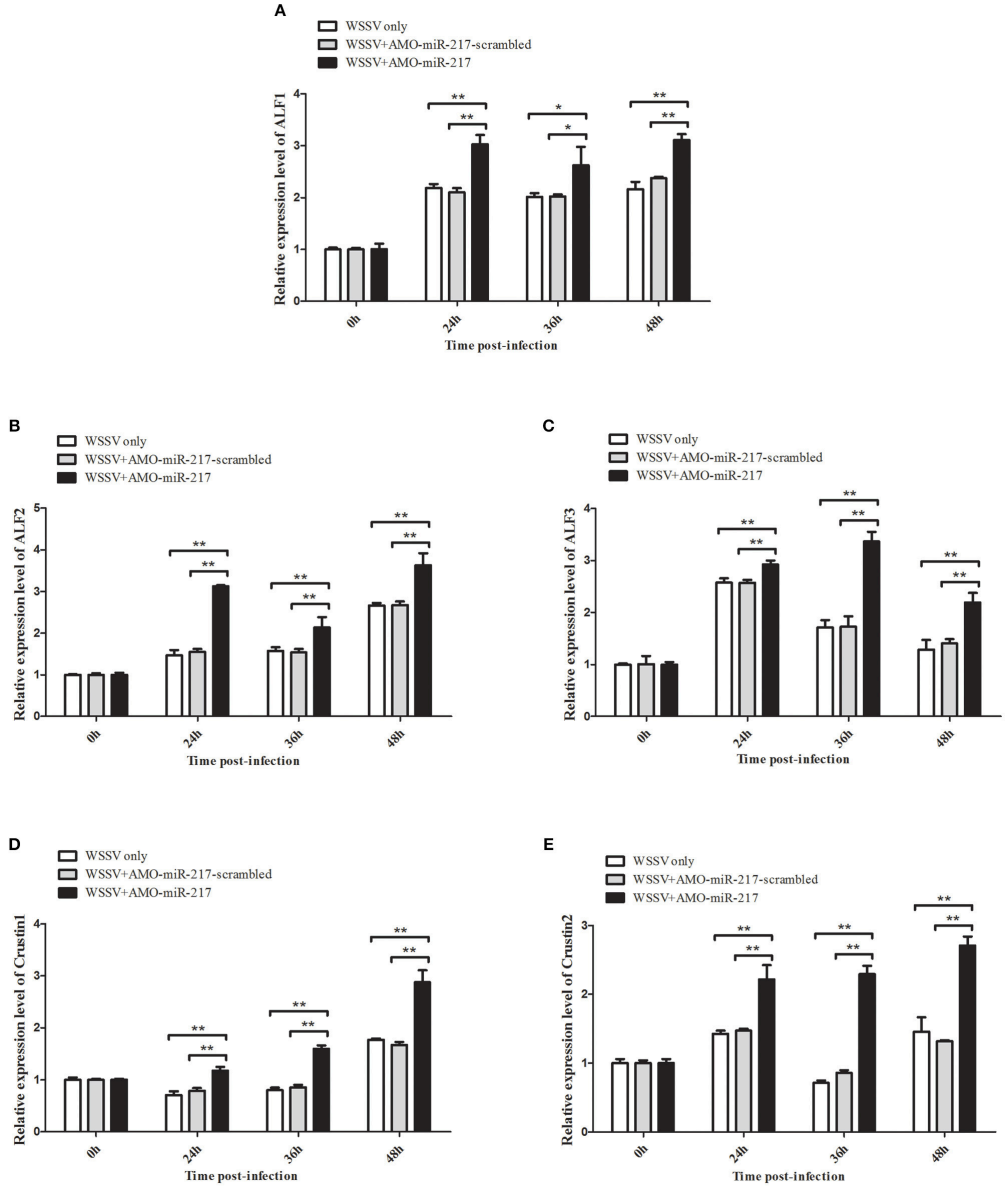
**Effects of miR-217 silencing on the transcriptions of five *AMPs in vivo***. AMO-miR-217 was injected into WSSV-infected crab to knock down the miR-217 expression. AMO-miR-217-scrambled, WSSV only were used as controls. At different time post-infection, the expression of *ALF1* **(A)**, *ALF2* **(B)**, *ALF3* **(C)**, *Crus1* **(D)**, or *Crus2* **(E)** were quantified by qRT-PCR. Statistically significant differences between treatments are indicated by asterisks (^*^p < 0.05; ^**^p < 0.01).

All the preceding results described implied that five *AMP* genes are the downstream effector genes regulated by *Tube*. Therefore, the pathway mediated by host miR-217 was further explored. The results indicated that the expression levels of *ALF1* (Figure 6A), *ALF2* (Figure 6B), *ALF3* (Figure 6C), *Crus1* (Figure 6D), and *Crus2* (Figure 6E) are significantly downregulated when miR-217 is overexpressed in crab. By contrast, the control miRNA injection had no effect on the transcription of these *AMP* genes. The knockdown of miR-217 by AMO-miR-217 led to a significant increase in the expression levels of *ALF1* (Figure 7A), *ALF2* (Figure 7B), *ALF3* (Figure 7C), *Crus1* (Figure 7D), and *Crus2* (Figure 7E) at 24 h to 48 h post-infection compared with those of WSSV only. By contrast, the control AMO-miR-217-scrambled had no effect on the transcriptions of *AMPs*, thereby suggesting that the host miR-217, *ALF1, ALF2, ALF3, Crus1*, and *Crus2* shared the same pathway.

### Expression of *ALF1, ALF2, ALF3, Crus1*, and *Crus2* during virus infection in crab

**Figure 8:**
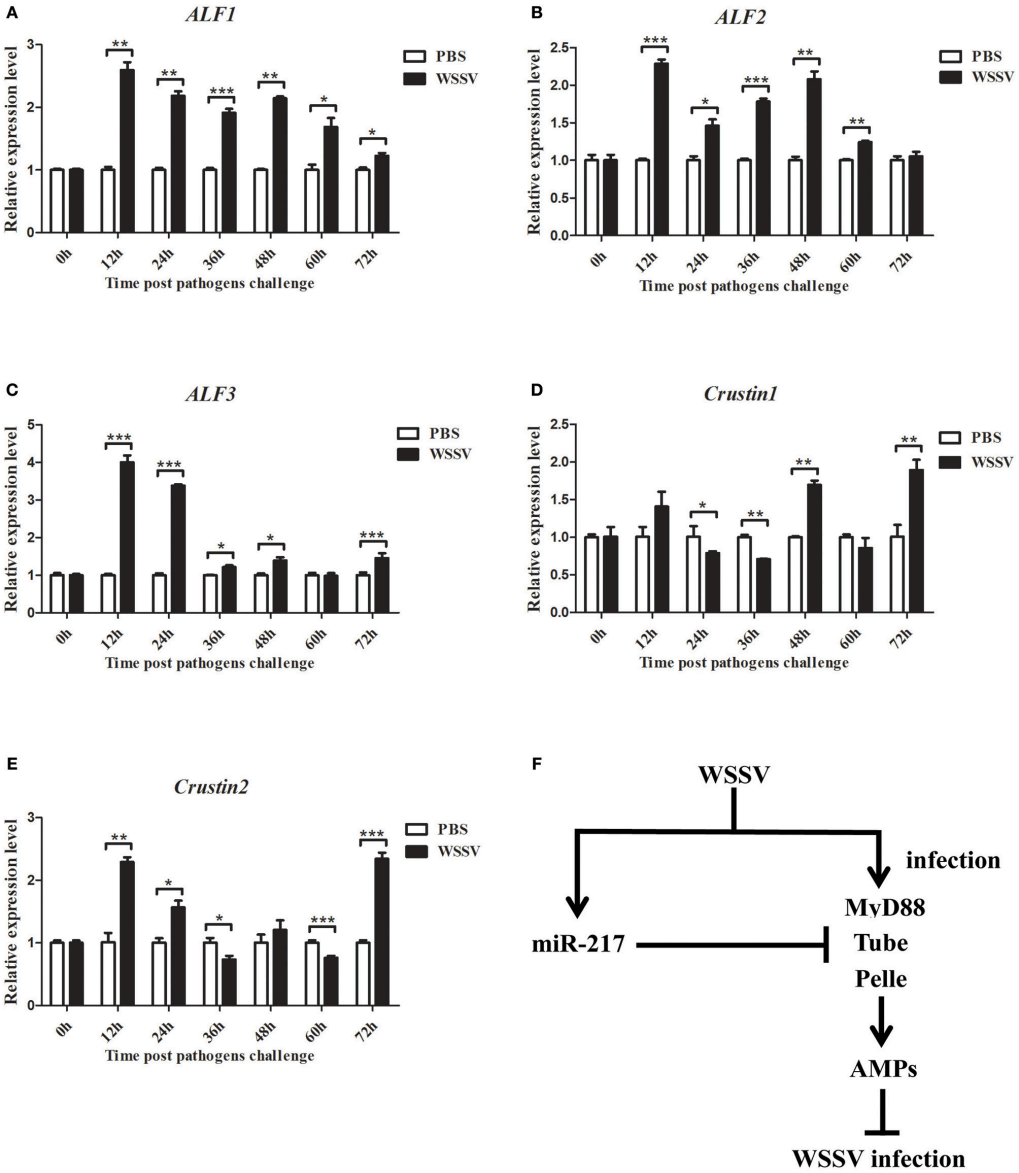
**Expression of *ALF1*, *ALF2*, *ALF3*, *Crus1*, and *Crus2* during virus infection in crab**. qRT-PCR method was used to analysis the expression of *ALF1* **(A)**, *ALF2* **(B)**, *ALF3* **(C)**, *Crus1* **(D)**, and *Crus2* **(E)** in the hemocytes of *E. sinensis* at 0, 12, 24, 36, 48, 60, and 72 h after WSSV challenge. The mRNA levels of these five AMPs were analyzed and standardized according to *GAPDH* mRNA levels. Asterisks indicate significant differences (^*^p < 0.05; ^**^p < 0.01; ^***^p < 0.001) compared with values of the control. Error bars represent ± S.D. of the three independent PCR amplifications and quantifications. **(F)** Model for the role of host miR-217 in virus-host interaction.

The genes of *ALF1, ALF2, ALF3, Crus1*, and *Crus2* were characterized and their expression patterns were performed in crabs to evaluate their roles in viral infection. As shown in Figures 8A–E, the mRNAs of *ALF1, ALF2, ALF3, Crus1*, and *Crus2* were upregulated post-WSSV infection at several time points. By contrast, the transcripts of the five *AMP* genes did not obviously change after PBS challenge. The data demonstrated that the five *AMP* genes might be involved in anti-viral innate immune defense. These findings together revealed that host miRNA (miR-217) inhibited the Toll signaling pathway by targeting the host *Tube* gene, resulting in the enhancement of virus replication in crabs (Figure 8F).

## Discussion

Although Toll signaling pathway has been implicated in the processes of virus infection, its function in host–virus interaction remains to be unfamiliar. MiRNAs are post-transcriptional regulatory molecules that could regulate gene expression and participate in numerous key biological processes by inhibiting mRNA translation or promoting mRNA decomposition (Meister and Tuschl, 2004; Bagga et al., 2005). Tumor-secreted miRNAs (miR-21 and miR-29a) can function as ligands, binding to the receptors of the Toll-like receptor (TLR) family, namely, murine *TLR7* and human *TLR8* (Fabbri et al., 2012). Human miR-9 was rapidly increased after *TLR4*-activation, which operated a feedback control of the NF-κB-dependent responses by fine-tuning the expression of a key member of the NF-κB family (Bazzoni et al., 2009). MiR-105 was reported as a modulator of *TLR2* translation in human gingival keratinocytes (Benakanakere et al., 2009). MiR-146 could control TLR and cytokine signaling through a negative feedback regulation loop involving the downregulation of IL-1 receptor-associated kinase 1 and TNF receptor-associated factor 6 protein levels (Taganov et al., 2006). However, miRNAs of crustaceans that mediate the regulation of key components of the host Toll pathway are still not well studied.

In the current study, we reported that crab-specific miR-217 played positive roles in WSSV infection by negatively regulating host Toll signaling pathway in *E. sinensis*. MiR-217 inhibited the Toll signaling pathway by targeting the *Tube* gene. Tube, which is the key component of host Toll pathway, played pivotal roles in numerous biological activities, such as survival, development, and innate immunity (Yu et al., 2014). The Tube could activate or repress a broad range of downstream genes and be involved in forming a sophisticated network in response to various stress signals. In many cases, an individual miRNA can target a unique gene and lead to subsequent changes of the biological processes associated with this target gene. The overexpression of crab miR-217 through the injection of miR-217-mimic led to the enhancement of WSSV copies, implying that miR-217 plays a positive role in WSSV replication. In the multinuclear-activation of galactosidase indicator cells, human miR-217 could influence the transactivation of Tat-induced human immunodeficiency virus 1 through the downregulation of *SIRT1* expression (Zhang et al., 2012). In crustaceans, host miRNAs were also found to be involved in virus infection. In *Marsupenaeus japonicas*, host miR-1000 was reported to contribute considerable effects on apoptosis and WSSV replication through targeting *p53* (Gong et al., 2015). Shrimp miR-7 could serve as an antiviral factor by interacting with viral *wsv477* (Huang and Zhang, 2012). In *Macrobrachium rosenbergii*, host miR-9041 and miR-9850 played positive roles in WSSV replication by targeting the *STAT* gene (Huang Y. et al., 2016). Our investigations revealed that crab miR-217 led to a decrease in the transcript level of the *Tube* gene, resulting in the enhancement of WSSV copies.

*AMP* genes are effector molecules in the innate immunity of invertebrates (Hancock et al., 2006). The *AMPs* of major crustaceans are represented by three cationic peptide families: *ALFs, crustins*, and *penaeidins* (Tassanakajon et al., 2010). *ALFs* are cationic *AMPs* with the ability to bind and neutralize lipopolysaccharide and are crucial bioactive substances in the innate immunity of crustaceans (Aketagawa et al., 1986). *Crustins* are cationic cysteine-rich *AMPs* (Liu et al., 2015). In *E. sinensis*, three *ALF* genes (*ALF1, ALF2*, and *ALF3*) and two *Crustin* genes (*Crus1* and *Crus2*) were identified. The upregulated expression of the five *AMP* genes in WSSV-challenged crabs may indicate their possible roles in anti-virus immune defense. *ALF* from *Fenneropeneaus chinensis* played a key role in inhibiting WSSV replication (Li S. et al., 2015). In *Penaeus monodon*, the enhanced crustin-like *AMP* conferred significant protection from WSSV infection (Antony et al., 2011). The knockdown of *Tube* gene resulted in a reduction of *ALF1, ALF2, ALF3, Crus1*, and *Crus2* transcripts in crabs. These results suggested that crab *AMP* genes could be modulated by the host Toll pathway, which is also the case in *Drosophila* (Lemaitre and Hoffmann, 2007). Similarly, in *Procambarus clarkii, PcToll* was involved in the regulation of *Crus* expression (Wang et al., 2015). The knockdown of *Dorsal* in *M. rosenbergii* resulted in the suppression of *Crus* transcripts (Huang X. et al., 2016). The silencing of the *PmMyD88* gene resulted in a decrease of expression of two *Crus* genes in *Litopenaeus vannamei* (Arayamethakorn et al., 2017). Therefore, we identified several *AMP* genes regulated by crab *tube* in our study. Further experiments indicated that overexpression of miR-217 inhibited the expression of these *AMP* genes. Hence, our findings together revealed that host miRNA (miR-217) targeting the host *tube* gene caused the inhibition of the expression of *AMP* genes, resulting in the enhancement of virus replication in crabs.

## Author contributions

YH, KH, and QR carried out the experiments. YH, WW, and QR designed the experiments and analyzed the data. YH and QR contributed reagents/materials. YH and QR wrote the manuscript. All authors gave final approval for publication.

## Funding

The current study was supported by the National Natural Science Foundation of China (Grant Nos. 31572647, 31272686), Natural Science Fund of Colleges and universities in Jiangsu Province (14KJA240002), High level talents in Nanjing Normal University Foundation (2012104XGQ0101), Graduate students Research and Innovation Program of Jiangsu Province (KYZZ15_0214), Jiangsu Agriculture Science and Technology Innovation Fund (CX(15)1011-02) and the Project Funded by the Priority Academic Program Development of Jiangsu Higher Education Institutions (PAPD).

### Conflict of interest statement

The authors declare that the research was conducted in the absence of any commercial or financial relationships that could be construed as a potential conflict of interest.

## References

[B1] Aketagawa J. Miyata T. Ohtsubo S. Nakamura T. Morita T. Hayashida H.. (1986). Primary structure of limulus anticoagulant anti-lipopolysaccharide factor. J. Biol. Chem. 261, 7357–7365. 3711091

[B2] Ambros V. (2004). The functions of animal microRNAs. Nature 431, 350–355. 10.1038/nature02871 15372042

[B3] Antony S. P. Singh I. S. Sudheer N. S. Vrinda S. Priyaja P. Philip R. (2011). Molecular characterization of a crustin-like antimicrobial peptide in the giant tiger shrimp, *Penaeus monodon*, and its expression profile in response to various immunostimulants and challenge with WSSV. Immunobiology 216, 184–194. 10.1016/j.imbio.2010.05.030 20580462

[B4] Arayamethakorn S. Supungul P. Tassanakajon A. Krusong K. (2017). Characterization of molecular properties and regulatory pathways of CrustinPm1 and CrustinPm7 from the black tiger shrimp *Penaeus monodon*. Dev. Comp. Immunol. 67, 18–29. 10.1016/j.dci.2016.10.015 27815179

[B5] Bagga S. Bracht J. Hunter S. Massirer K. Holtz J. Eachus R.. (2005). Regulation by let-7 and lin-4 miRNAs results in target mRNA degradation. Cell 122, 553–563. 10.1016/j.cell.2005.07.031 16122423

[B6] Bartel D. P. (2004). MicroRNAs: genomics, biogenesis, mechanism, and function. Cell 116, 281–297. 10.1016/S0092-8674(04)00045-5 14744438

[B7] Bazzoni F. Rossato M. Fabbri M. Gaudiosi D. Mirolo M. Mori L.. (2009). Induction and regulatory function of miR-9 in human monocytes and neutrophils exposed to proinflammatory signals. Proc. Natl. Acad. Sci. U.S.A. 106, 5282–5287. 10.1073/pnas.0810909106 19289835 PMC2664036

[B8] Belvin M. P. Anderson K. V. (1996). A conserved signaling pathway: the Drosophila toll-dorsal pathway. Annu. Rev. Cell Dev. Biol. 12, 393–416. 10.1146/annurev.cellbio.12.1.393 8970732

[B9] Benakanakere M. R. Li Q. Eskan M. A. Singh A. V. Zhao J. Galicia J. C.. (2009). Modulation of TLR2 protein expression by miR-105 in human oral keratinocytes. J. Biol. Chem. 284, 23107–23115. 10.1074/jbc.M109.013862 19509287 PMC2755716

[B10] Bernstein E. Caudy A. A. Hammond S. M. Hannon G. J. (2001). Role for a bidentate ribonuclease in the initiation step of RNA interference. Nature 409, 363–366. 10.1038/35053110 11201747

[B11] Bischoff V. Vignal C. Boneca I. G. Michel T. Hoffmann J. A. Royet J. (2004). Function of the Drosophila pattern-recognition receptor PGRP-SD in the detection of gram positive bacteria. Nat. Immunol. 5, 1175–1180. 10.1038/ni1123 15448690

[B12] Carrington J. C. Ambros V. (2003). Role of microRNAs in plant and animal development. Science 301, 336–338. 10.1126/science.1085242 12869753

[B13] Ding Z. Yao Y. Zhang F. Wan J. Sun M. Liu H. (2015). The first detection of white spot syndrome virus in naturally infected cultured Chinese mitten crabs, *Eriocheir sinensis* in China. J. Virol. Methods 220, 49–54. 10.1016/j.jviromet.2015.04.011 25907468

[B14] Escobedo-Bonilla C. M. Alday-Sanz V. Wille M. Sorgeloos P. Pensaert M. B. Nauwynck H. J. (2008). A review on the morphology, molecular characterization, morphogenesis and pathogenesis of white spot syndrome virus. J. Fish Dis. 31, 1–18. 10.1111/j.1365-2761.2007.00877.x 18086030

[B15] Fabbri M. Paone A. Calore F. Galli R. Gaudio E. Santhanam R.. (2012). MicroRNAs bind to Toll-like receptors to induce prometastatic inflammatory response. Proc. Natl. Acad. Sci. U.S.A. 109, E2110–E2116. 10.1073/pnas.1209414109 22753494 PMC3412003

[B16] Gong Y. Ju C. Zhang X. (2015). The miR-1000-p53 pathway regulates apoptosis and virus infection in shrimp. Fish Shellfish Immunol. 46, 516–522. 10.1016/j.fsi.2015.07.022 26220644

[B17] Hancock R. E. Brown K. L. Mookherjee N. (2006). Host defence peptides from invertebrates-emerging antimicrobial strategies. Immunobiology 211, 315–322. 10.1016/j.imbio.2005.10.017 16697922

[B18] Huang T. Zhang X. (2012). Functional analysis of a crustacean microRNA in host-virus interactions. J. Virol. 86, 12997–13004. 10.1128/JVI.01702-12 23015693 PMC3497620

[B19] Huang X. Wang W. Ren Q. (2016). Dorsal transcription factor is involved in regulating expression of crustin genes during white spot syndrome virus infection. Dev. Comp. Immunol. 63, 18–26. 10.1016/j.dci.2016.05.006 27181712

[B20] Huang Y. Wang W. Ren Q. (2016). Two host microRNAs influence WSSV replication via STAT gene regulation. Sci. Rep. 6:23643. 10.1038/srep23643 27029712 PMC4814834

[B21] Imler J. L. Hoffmann J. A. (2001). Toll receptors in innate immunity. Trends Cell Biol. 11, 304–311. 10.1016/S0962-8924(01)02004-9 11413042

[B22] Kawai T. Akira S. (2010). The role of pattern-recognition receptors in innate immunity: update on Toll-like receptors. Nat. Immunol. 11, 373–384. 10.1038/ni.1863 20404851

[B23] Lecellier C. H. Dunoyer P. Arar K. Lehmann-Che J. Eyquem S. Himber C.. (2005). A cellular microRNA mediates antiviral defense in human cells. Science 308, 557–560. 10.1126/science.1108784 15845854

[B24] Lee R. C. Feinbaum R. L. Ambros V. (1993). The, *C. elegans* heterochronic gene lin-4 encodes small RNAs with antisense complementarity to lin-14. Cell 75, 843–854. 10.1016/0092-8674(93)90529-Y 8252621

[B25] Lemaitre B. Hoffmann J. (2007). The host defense of Drosophila melanogaster. Annu. Rev. Immunol. 25, 697–743. 10.1146/annurev.immunol.25.022106.141615 17201680

[B26] Lemaitre B. Nicolas E. Michaut L. Reichhart J. M. Hoffmann J. A. (1996). The dorsoventral regulatory gene cassette spätzle/Toll/cactus controls the potent antifungal response in Drosophila adults. Cell 86, 973–983. 10.1016/S0092-8674(00)80172-5 8808632

[B27] Li L. Wei Z. Zhou Y. Gao F. Jiang Y. Yu L.. (2015). Host miR-26a suppresses replication of porcine reproductive and respiratory syndrome virus by upregulating type I interferons. Virus Res. 195, 86–94. 10.1016/j.virusres.2014.08.012 25218480 PMC7114497

[B28] Li S. Guo S. Li F. Xiang J. (2015). Functional diversity of anti-lipopolysaccharide factor isoforms in shrimp and their characters related to antiviral activity. Mar. Drugs 13, 2602–2616. 10.3390/md13052602 25923317 PMC4446596

[B29] Li X. C. Zhu L. Li L. G. Ren Q. Huang Y. Q. Lu J. X.. (2013). A novel myeloid differentiation factor 88 homolog, SpMyD88, exhibiting SpToll binding activity in the mud crab *Scylla paramamosain*. Dev. Comp. Immunol. 39, 313–322. 10.1016/j.dci.2012.11.011 23280154

[B30] Liu N. Lan J. F. Sun J. J. Jia W. M. Zhao X. F. Wang J. X. (2015). A novel crustin from *Marsupenaeus japonicus* promotes hemocyte phagocytosis. Dev. Comp. Immunol. 49, 313–322. 10.1016/j.dci.2014.11.021 25479014

[B31] Liu W. Han F. Zhang X. (2009). Ran GTPase regulates hemocytic phagocytosis of shrimp by interaction with myosin. J. Proteome Res. 8, 1198–1206. 10.1021/pr800840x 19166347

[B32] Livak K. J. Schmittgen T. D. (2001). Analysis of relative gene expression data using real-time quantitative PCR and the 2^−ΔΔCT^ method. Methods 25, 402–408. 10.1006/meth.2001.1262 11846609

[B33] Meister G. Tuschl T. (2004). Mechanisms of gene silencing by double-stranded RNA. Nature 431, 343–349. 10.1038/nature02873 15372041

[B34] Mosca N. Castiello F. Coppola N. Trotta M. C. Sagnelli C. Pisaturo M.. (2014). Functional interplay between hepatitis B virus X protein and human miR-125a in HBV infection. Biochem. Biophys. Res. Commun. 449, 141–145. 10.1016/j.bbrc.2014.05.009 24824183

[B35] Pareek S. Roy S. Kumari B. Jain P. Banerjee A. Vrati S. (2014). MiR-155 induction in microglial cells suppresses Japanese encephalitis virus replication and negatively modulates innate immune responses. J. Neuroinflammation 11:97. 10.1186/1742-2094-11-97 24885259 PMC4050406

[B36] Pedersen I. M. Cheng G. Wieland S. Volinia S. Croce C. M. Chisari F. V. (2007). Interferon modulation of cellular microRNAs as an antiviral mechanism. Nature 499, 919–922. 10.1038/nature06205 PMC2748825 17943132

[B37] Reinhart B. J. Slack F. J. Basson M. Pasquinelli A. E. Bettinger J. C. Rougvie A. E.. (2000). The 21-nucleotide let-7 RNA regulates developmental timing in *Caenorhabditis elegans*. Nature 403, 901–906. 10.1038/35002607 10706289

[B38] Schwarz D. S. Hutvágner G. Du T. Xu Z. Aronin N. Zamore P. D. (2003). Asymmetry in the assembly of the RNAi enzyme complex. Cell 115, 199–208. 10.1016/S0092-8674(03)00759-1 14567917

[B39] Sun J. Z. Wang J. Wang S. Yuan D. Li Z. Yi B.. (2014). MicroRNA miR-320a and miR-140 inhibit mink enteritis virus infection by repression of its receptor, feline transferrin receptor. Virol. J. 11, 210. 10.1186/s12985-014-0210-3 25465595 PMC4264318

[B40] Taganov K. D. Boldin M. P. Chang K. J. Baltimore D. (2006). NF-κB-dependent induction of microRNA miR-146, an inhibitor targeted to signaling proteins of innate immune responses. Proc. Natl. Acad. Sci. U.S.A. 103, 12481–12486. 10.1073/pnas.0605298103 16885212 PMC1567904

[B41] Tassanakajon A. Amparyup P. Somboonwiwat K. Supungul P. (2010). Cationic antimicrobial peptides in penaeid shrimp. Mar. Biotechnol. 12, 487–505. 10.1007/s10126-010-9288-9 20379756

[B42] Triboulet R. Mari B. Lin Y. L. Chable-Bessia C. Bennasser Y. Lebrigand K.. (2007). Suppression of microRNA-silencing pathway by HIV-1 during virus replication. Science 315, 1579–1582. 10.1126/science.1136319 17322031

[B43] Uematsu S. Akira S. (2008). Toll-like receptors (TLRs) and their ligands. Handb. Exp. Pharmacol. 1–20. 10.1007/978-3-540-72167-3_1 18071652

[B44] Umbach J. L. Cullen B. R. (2009). The role of RNAi and microRNAs in animal virus replication and antiviral immunity. Genes Dev. 23, 1151–1164. 10.1101/gad.1793309 19451215 PMC2763533

[B45] Valanne S. Wang J. H. Rämet M. (2011). The Drosophila toll signaling pathway. J. Immunol. 186, 649–656. 10.4049/jimmunol.1002302 21209287

[B46] Wang Z. Chen Y. H. Dai Y. J. Tan J. M. Huang Y. Lan J. F.. (2015). A novel vertebrates Toll-like receptor counterpart regulating the anti-microbial peptides expression in the freshwater crayfish, *Procambarus clarkii*. Fish Shellfish Immunol. 43, 219–229. 10.1016/j.fsi.2014.12.038 25573502

[B47] Wang Z. Liu J. Sudom A. Ayres M. Li S. Wesche H.. (2006). Crystal structures of IRAK-4 kinase in complex with inhibitors: a serine/threonine kinase with tyrosine as a gatekeeper. Structure 14, 1835–1844. 10.1016/j.str.2006.11.001 17161373

[B48] Yu A. Q. Jin X. K. Wu M. H. Guo X. N. Li S. He L.. (2014). Identification and characterization of Tube in the Chinese mitten crab *Eriocheir sinensis*. Gene 541, 41–50. 10.1016/j.gene.2014.03.009 24630961

[B49] Zhang H. S. Wu T. C. Sang W. W. Ruan Z. (2012). MiR-217 is involved in Tat-induced HIV-1 long terminal repeat (LTR) transactivation by downregulation of SIRT1. Biochim. Biophys. Acta 1823, 1017–1023. 10.1016/j.bbamcr.2012.02.014 22406815

